# Reliability and advantages of using non-uniform Chang’s attenuation correction method using a CT-based attenuation coefficient map in ^99m^Tc-GSA SPECT/CT hepatic imaging

**DOI:** 10.1186/s40658-015-0120-5

**Published:** 2015-08-14

**Authors:** Yuya Nakamura, Seiji Tomiguchi, Masayuki Tanaka

**Affiliations:** Division of Medical Technology, Kumamoto University Hospital, 1-1-1 Honjyo, Chuo-ku, Kumamoto City, 860-8556 Japan; Graduate School of Health Sciences, Kumamoto University, 4-24-1, Kuhonji, Chuo-ku, Kumamoto City, 862-0976 Japan; Faculty of Life Sciences, Kumamoto University, 4-24-1, Kuhonji, Chuo-ku, Kumamoto City, 862-0976 Japan

**Keywords:** ^99m^Tc-GSA SPECT/CT, Attenuation correction, Non-uniform Chang’s AC

## Abstract

**Background:**

Generally, attenuation correction is made by incorporating a linear attenuation coefficient, which is based on the attenuation coefficient map (mu-map) created from a computed tomography scan, into the ordered subsets-expectation maximization reconstruction method in non-uniform domains. A non-uniform Chang’s attenuation correction method that uses the mu-map created from a computed tomography image that was made after reconstruction has been performed is currently available. The purpose of this study was to determine the usefulness of the non-uniform Chang’s attenuation correction method in ^99m^Tc-galactosyl human serum albumin diethylenetriamine pentaacetic acid single photon emission computed tomography/computed tomography imaging.

**Methods:**

Single photon emission computed tomography/computed tomography imaging was performed in phantoms with ^99m^Tc water solutions and in a clinical study of 20 donors in living liver tissue transplantation. Attenuation correction was then performed in the reconstructed single photon emission computed tomography images with the non-uniform Chang’s method and ordered subsets-expectation maximization attenuation correction methods with triple energy window scatter correction. Root mean square error values were used for assessment of the image uniformity, and we evaluated the absolute radioactivity in liver parts in the phantoms and those in the donors who had a normal liver function.

**Results:**

The values of root mean square error on the non-uniform Chang’s attenuation correction images were lower than those on ordered subsets-expectation maximization attenuation correction images for both the phantoms and the 20 donors. The difference between the true and estimated radioactivity in the non-uniform Chang’s attenuation correction method was smaller than that in the ordered subsets-expectation maximization attenuation correction methods in the phantom study.

**Conclusions:**

The non-uniform Chang’s attenuation correction is considered to be superior to the ordered subsets-expectation maximization attenuation correction in the assessment of absolute liver radioactivity and liver image uniformity on ^99m^Tc-galactosyl human serum albumin diethylenetriamine pentaacetic acid single photon emission computed tomography/computed tomography imaging.

## Introduction

^99m^Tc-galactosyl human serum albumin diethylenetriamine pentaacetic acid (GSA) dynamic planner imaging is useful for the evaluation of hepatic functional reserve in the whole liver in cases such those with chronic liver diseases [1]. However, it is difficult to evaluate regional hepatic functional reserve including the regional volume. Therefore, single photon emission computed tomography (SPECT) imaging is necessary to evaluate the regional hepatic functional reserve.

^99m^Tc-GSA SPECT/computed tomography (CT) imaging is used for evaluation of the regional hepatic functional reserve. Many quantitative indices for the regional hepatic functional reserve have been applied clinically [2, 3]. The liver functional image in three dimensions is obtained by SPECT imaging at the moment when the plateau of the liver accumulation of GSA is reached, just before it is excreted into the biliary tracts [4]. For evaluation of the exact GSA liver uptake quantitatively, attenuation correction (AC) with a high precision gamma-ray is required. Because the accumulation of GSA in normal hepatocytes for an injection dose is relatively high, the frequent appearance of a streak artifact is an intractable problem in the quantitative assessment of the hepatic function by the use of a filtered back projection (FBP) algorithm when image reconstruction (FBP reconstruction) is used. Thus, it is important to prevent the appearance of unwelcome streak artifacts in the reconstructed images in FBP reconstructions as much as possible. On the other hand, the noise in the SPECT image increases relatively in patients having a severely deteriorated liver function because of the low liver uptake of GSA. In such patients, using the maximum likelihood-expectation maximization (ML-EM) [5] or the ordered subsets-expectation maximization (OS-EM) algorithm [6] is helpful in image reconstruction for controlling the noise.

An attenuation correction is usually performed by incorporating of the linear attenuation coefficients (mu-value) into the detection probability acquired from the ML-EM and OS-EM reconstruction methods. Recently, an OS-EM reconstruction method including the AC (OS-EM AC method) has been gaining wider use because of its rapid reconstruction speed [7].

However, there is a problem of the quality of the reconstructed image being affected by a combination of the number of iterations and subsets (reconstruction parameter) [8]. Although it is desirable to determine the reconstruction parameter that is optimal for every patient, nevertheless the same reconstruction parameter is generally and constantly employed in clinical treatment. As a result, either an underestimation or an overestimation of an organ’s radioactivity frequently occurs in the quantitative assessment process. Also, the accuracy of the AC is affected by the number of iterations and subsets because the AC has been performed within the iterative reconstruction process. Furthermore, the optimal iteration numbers and subsets have not been adequately defined in cases where the AC was performed according to OS-EM algorithms.

The AC method for uniform activity and μ (linear attenuation coefficient) distribution started with the description by Sorenson [9], which was then improved by Chang [10] for non-uniform activity distribution and later was further enhanced for both non-uniform activity and μ distribution by Wu et al. [11]. Chang’s AC technique has been widely used for brain. However, several new Chang’s AC methods based on the mu-map (non-uniform Chang’s AC method) are now available for clinical use [11, 12]. These methods also enable attenuation correction for most non-uniform organs.

The non-uniform Chang’s AC method consists of the following procedures. First, a CT value is acquired from the CT image. Second, the CT value is converted into a mu-value using the bilinear scale method. For the conversion of Hounsfield unit (HU) to a mu-value, a transition point of the HU in the bilinear scale was chosen at 0 HU. Thirdly, the mu-value of each pixel is assigned to the general formula of the non-uniform Chang’s AC method. Finally, the AC is carried out after completion of scatter corrected SPECT image reconstruction using the general formula of the non-uniform Chang’s AC method (2nd order).

Scatter correction (SC) for SPECT data is necessary with AC because the mu-value calculated from this method is for narrow beams. A triple energy window (TEW) [13] method was used in the present study.

The non-uniform Chang’s AC method was adopted for ^99m^Tc-GSA SPECT/CT imaging tests in this study. The usefulness of the method was then evaluated in both phantoms and patients. In addition, the probable number of iterations that affect the attenuation corrected image was calculated.

## Materials and methods

### Phantom and donors

For the phantom study, the liver phantom LKS (Kyoto Kagaku Co., Ltd.) was used. A liver part in this phantom was filled with a water solution of ^99m^Tc. The high (160 MBq of ^99m^Tc) and low (80 MBq of ^99m^Tc) radioactivity concentrations in the liver areas were filled.

For the donors’ study, a retrospective study from June 2012 to December 2012 was approved by our institutional review board; informed consent was obtained from all donors before the start of the SPECT/CT study [14]. A total of 20 donors for liver tissue transplantation were enrolled. They consisted of 10 men and 10 women. The subjects’ age ranged from 20 to 67 years (mean age 42.3 years). A dose of 185 MBq of ^99m^Tc-GSA was injected as a bolus into the antecubital vein in each donor.

### SPECT/CT imaging

SPECT/CT imaging was performed with a two-detector hybrid-type SPECT/CT scanner (Symbia T16^™^, SIEMENS) equipped with a low-energy high-resolution (LEHR) collimator. SPECT data were acquired for 12 min with 1.23 magnifications, 128 × 128 matrixes (3.3 mm pixel size), 360° acquisition arc, 90 projections, and the continuous mode of the automatic proximity orbit. The acquisition window widths were 20 % for the main window at 140 keV (126–154 keV) of ^99m^Tc and 7 % for the lower sub-window at 122 keV (117–126 keV) [15]. A sub-window was set for the TEW scatter correction method.

Non-contrast-enhanced helical CT images were obtained with the same SPECT/CT system. The scanning parameters for all phases were as follows: tube voltage, 110 kVp; tube current, 50 mA; rotation time, 0.8 s; beam collimation, 1.25 mm; beam pitch, 0.5; field of view, 300 mm; and matrix, 512 × 512. After the initial registration of the SPECT and CT images, a mu-map was created. The mu-value was derived from the CT value through the bilinear conversion method as previously reported [16].

Image reconstruction was performed with the OS-EM method using the Syngo Acquisition Work Space^™^ (SIEMENS). The number of iterations was changed in the reconstruction processing with 1, 2, 3, 4, 5, 6, 7, 8, 9, 10, 15, 20, 25, and 30 (the subset number was fixed at 6). A Butterworth filter (cutoff value, 0.65 cycles/cm; order, 8/clinical recommended parameters) was used as a pre-filter. Both AC and SC were applied on the reconstructed images. However, resolution recovery was not used for the reconstruction. A Syngo Acquisition Work Space^™^ (SIEMENS) was used for both the AC and SC trials. Images of both OS-EM AC and non-uniform Chang’s AC were reconstructed for evaluation. Non-uniform Chang’s AC was performed after the completion of the SPECT image reconstruction according to the OS-EM algorithm.

### Quantitative indices (uniformity)

**Fig. 1:**
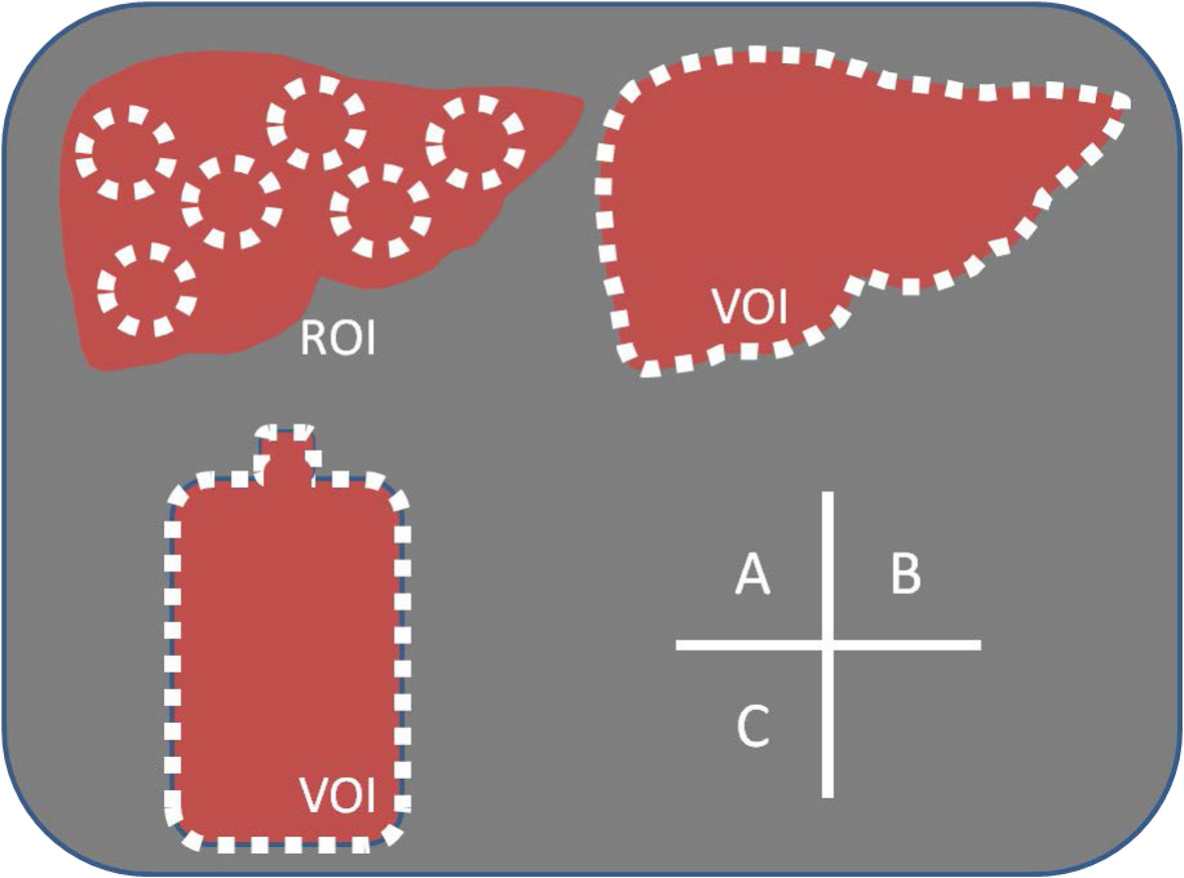
ROI and VOI used in the present study. They are ROIs for calculating RMSE (*A*), VOI for obtaining absolute radioactivity (*B*), and VOI for calculating CCF (*C*)

Six regions of interest (ROIs) were delineated in the liver part of the phantom or in a donor’s liver on the reconstructed SPECT images (Fig. 1(A)). The root mean square error (RMSE) value (1) from a mean count and its standard deviation in all ROIs was then calculated as the index for defining uniformity. The formula used for calculating the RMSE was as follows;

1 $$ \mathrm{RMSE} = \mathrm{S}\mathrm{D}/\mathrm{mean}\times 100\ \left(\%\right) $$

where mean is the mean counts and SD is the standard deviation.

### Quantitative indices (absolute radioactivity)

For assessment of the absolute radioactivity in a liver part of the phantom and the donor’s whole liver, a volume of interest (VOI) was set for the liver or a particular liver area thereof (Fig. 1(B)). The absolute radioactivity in the VOI was obtained by conversion of the total counts into the radioactivity in the VOI by the use of cross calibration factors (CCF). A half-life correction was also performed in the calculation of the radioactivity in the VOI. The formula for the absolute radioactivity was as follows;

2 $$ \mathrm{Radioactivity}\ \left(\mathrm{MBq}\right) = \mathrm{C}\mathrm{C}\mathrm{F}\times \mathrm{total}\ \mathrm{counts} $$

Six different radioactivities in a bottle phantom were used for calculation of the CCF. They were 1, 1/2, 1/4, 1/8, 1/16, and 1/32 of the ^99m^Tc solutions, with a maximum radioactivity of 400 MBq in the bottle phantom. SPECT imaging was performed, and the reconstructing was done according to the methods previously described. Then, a VOI was set for the whole bottle (Fig. 1(C)), and the total number of counts in the VOI was measured in each of the SPECT images. The true radioactivity in each bottle phantom was also measured with a well-type scintillation counter.

In this study, the mean values of CCF in each bottle phantom were defined as the CCF. The CCF was calculated for every iteration number. The estimated radioactivity in the VOI of the liver part or the entire liver was compared with the true radioactivity measured by a well-type scintillation counter.

### Evaluations

The RMSE as well as the estimated absolute radioactivity in liver parts or the whole liver were investigated as standard procedures. The optimal number of iterations was derived from the quantitative assessment of RMSE values and the absolute radioactivity in liver parts in the phantom study, together with detailed examination of the livers of the 20 donors. The optimal number of iterations was then used for the reconstruction of the SPECT data for all donors enrolled in this study. After that, comparisons between OS-EM AC images and non-uniform Chang’s AC scans were performed.

## Results

### RMSE and estimated radioactivity in the phantom study and the 20 donors

**Fig. 2:**
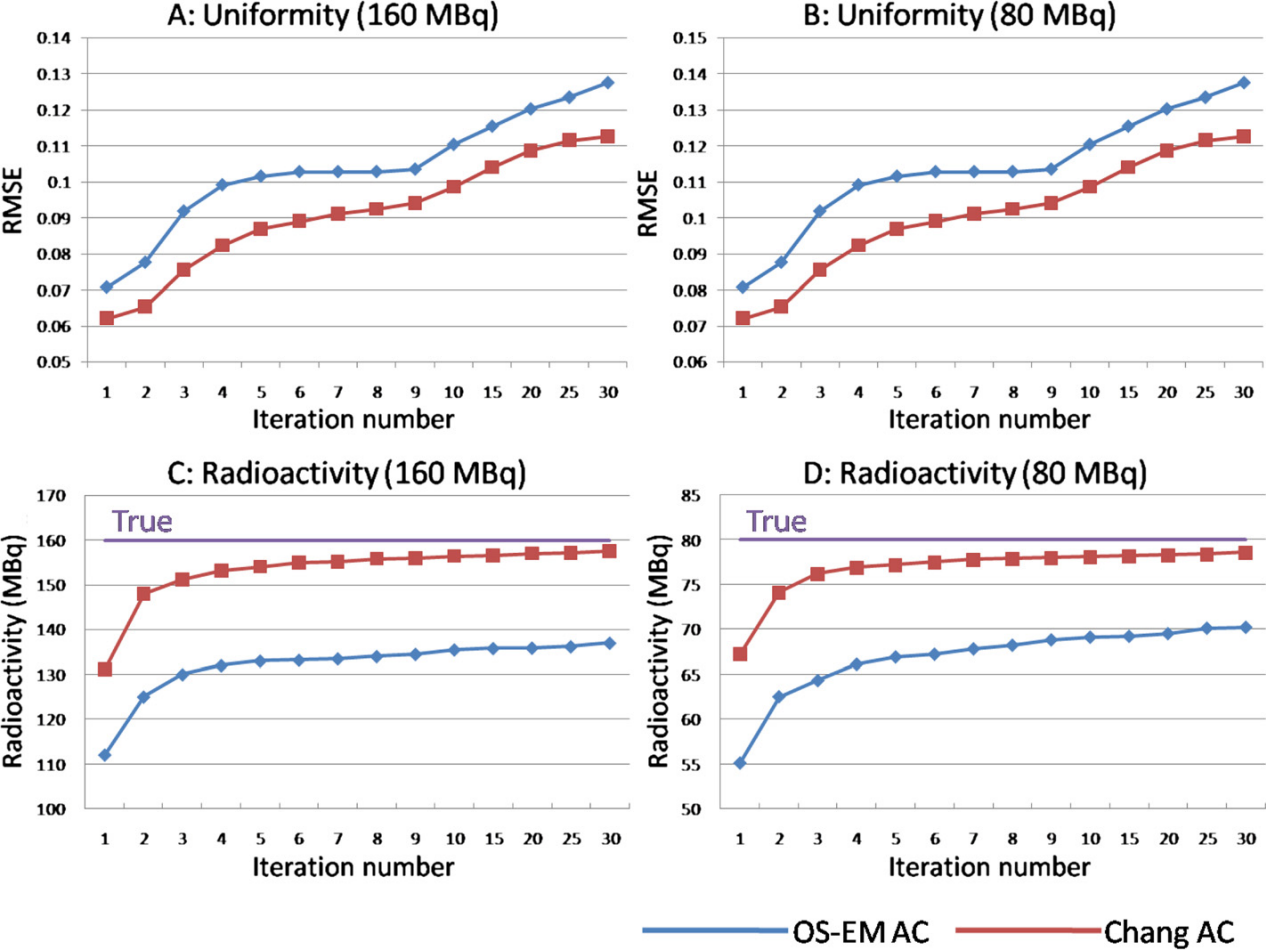
Uniformity (**a**, **b**) and estimated radioactivity (**c**, **d**) in the phantom study

**Fig. 3:**
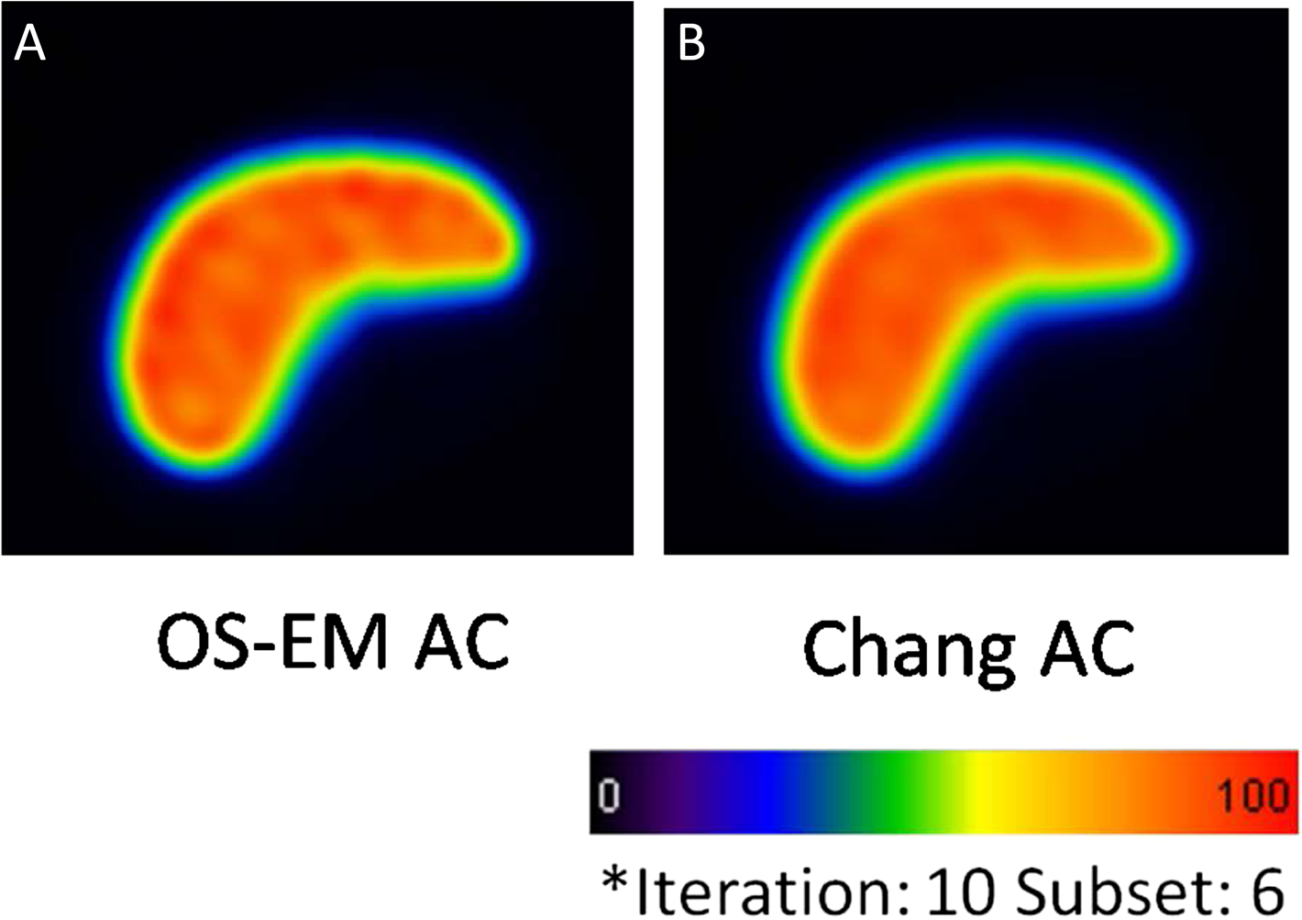
Representative reconstructed images of by OS-EM AC (**a**) and Chang AC (**b**)

**Fig. 4:**
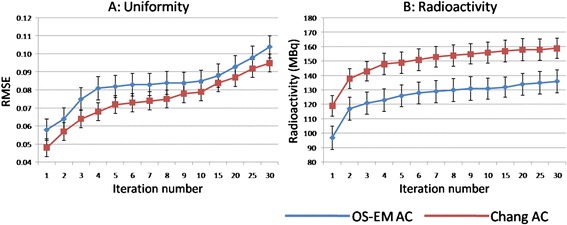
Uniformity (**a**) and estimated radioactivity (**b**) in the volunteer patient study

The values of RMSE and the difference between the true radioactivity and estimated radioactivity in a liver part corresponding to the number of iterations are indicated in Fig. 2. The RMSE values in the liver parts of the 160 MBq were lower than those of the 80 MBq in each iteration (Fig. 2a, b). As the number of iterations increased, the RMSE values also became higher for both phantoms. The values of RMSE in non-uniform Chang’s AC images were lower than those in the OS-EM AC images for both phantoms, with different radioactivity. On the other hand, the estimated radioactivity in a liver part reached a plateau at the 6th iteration in both phantoms when the non-uniform Chang’s AC technique (Fig. 2c, d) was used. However, the estimated radioactivity rose with an increase in the number of iterations with the OS-EM AC method (Fig. 2c, d). The difference between the true and the estimated radioactivity in non-uniform Chang’s AC method was smaller than that in the OS-EM AC method for both phantoms. In addition, the uniformity in the liver part on a non-uniform Chang’s AC (Fig. 3b) image was visually superior to that on the OS-EM AC image (Fig. 3a). The mean values and standard deviations (SDs) of the RMSE in the livers of the 20 donors showed a tendency similar to that for those obtained in the phantom studies (Fig. 4a). The estimated radioactivity in the liver also reached a plateau at six iterations in the 20 donors for the non-uniform Chang AC method (Fig. 4b). Therefore, six is considered to be the optimal number of iterations.

## Discussion

Attenuation correction is valuable for the quantitation of absolute organ radioactivity in SPECT imaging.

The non-uniform Chang’s AC technique proved superior to OS-EM AC methods for uniformity in parts of the liver investigated in the phantom study. The noise in the reconstructed SPECT image grew with an increase in the number of iterations when an OS-EM algorithm was used [17, 18]. OS-EM algorithms are used in both non-uniform Chang’s AC and OS-EM AC methods, and the same mu-map is used. The attenuation correction is performed in the iterative reconstruction process with OS-EM AC methods. However, with non-uniform Chang’s AC method, the correction is carried out after completion of the OS-EM reconstruction of the scatter corrected SPECT data. The increase in noise may influence the regional mu-value when the OS-EM AC method is used. On the other hand, regional mu-value is not affected by the non-uniform Chang’s AC method because the AC is not carried out in the iterative reconstruction process. This is considered to be one of the main reasons why the uniformity of the non-uniform Chang’s AC images was superior to that of the OS-EM AC images in this study.

Non-uniform Chang’s AC method also proved superior to the OS-EM AC methods in the evaluation of the quantitative accuracy in liver parts. In this study, the estimated radioactivity in part of the liver reached a plateau at six iterations on the non-uniform Chang’s AC images. On the other hand, it increased with the increase in the number of iterations on the OS-EM AC images. Therefore, a larger number of iterations are necessary for improving the quantitative accuracy for part of the liver with the OS-EM AC method. As many as 30 iterations or more with the OS-EM AC method would seem necessary for obtaining the same quantitative accuracy in a liver part as that which is indicated by the non-uniform Chang’s AC method. However, a large number of iterations results in inferior image quality because of the increase in noise. Therefore, the non-uniform Chang’s AC method is considered to be much more advantageous than the OS-EM AC method for the quantification of absolute radioactivity in the liver.

In the present study, it was difficult to define the optimal number of iterations for the OS-EM AC because the estimated radioactivity in the liver part and the whole liver increased with an increase in the number of iterations. Therefore, the optimal number of iterations was defined as being six, based on the results of the non-uniform Chang’s AC method in the phantom and the donors’ study. The non-uniform Chang’s AC images with six iterations showed a reasonable estimated radioactivity in liver parts of the phantom, with a relatively low RMSE value. The same trend could be observed in the donors.

There are a few limitations to this study. First, the optimal number for the subsets was not investigated. Further research is necessary for determining the optimal combination of the exact number of iterations and subsets in both the non-uniform Chang’s AC and the OS-EM AC methods. Second, the number of donors examined may have been too small for definitive clinical evaluations. More clinical studies with a larger number of patients are necessary for fully demonstrating the usefulness of using the non-uniform Chang’s AC in ^99m^Tc-GSA SPECT/CT imaging.

## Conclusion

Based on our examinations, the non-uniform Chang’s AC method is considered to be superior to the OS-EM AC method in the assessment of absolute liver radioactivity and liver image uniformity on ^99m^Tc-GSA SPECT/CT imaging.
